# Single and Multiobjective Shutdown Optimization of a Multistage Continuous Crystallizer

**DOI:** 10.1021/acs.iecr.3c03441

**Published:** 2024-04-09

**Authors:** Jiaxu Liu, Brahim Benyahia

**Affiliations:** Chemical Engineering Department, Loughborough University, Epinal Way, Loughborough, Leicestershire LE11 3TU, U.K.

## Abstract

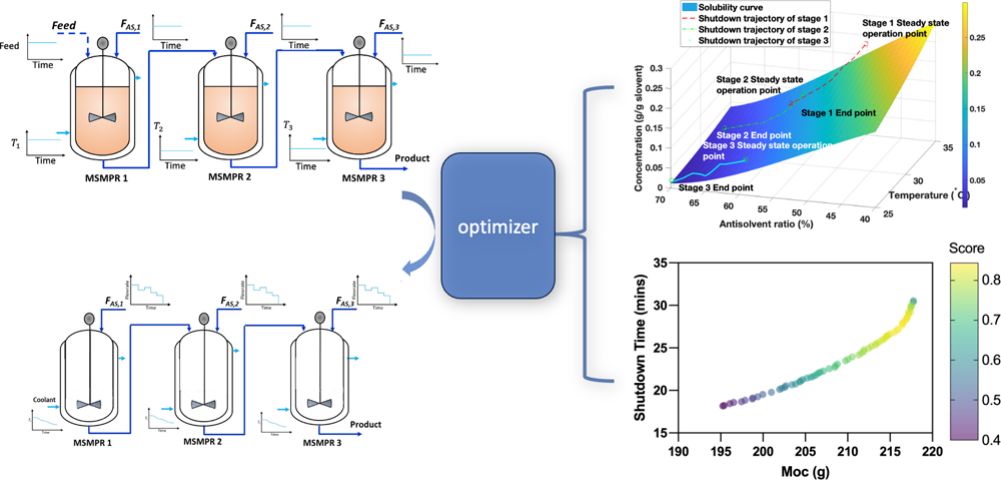

This study presents the first model-based optimal shutdown procedure of a multistage continuous crystallization process which aims at the maximization of on-spec production and minimization of the shutdown time. The cooling antisolvent crystallization of Aspirin (acetylsalicylic acid) in a three-stage continuous crystallizer was used as a case study. To address the optimal shutdown problem, several single optimization scenarios were considered to assess the impact of the degrees of freedom, discretization schemes, and optimization settings such as the constraints. The proposed optimal shutdown procedures showed that significant amounts of on-spec crystals can be produced both at fixed and variable shutdown times. Most importantly, the optimal shutdown procedures can match the steady-state productivity, based on the shutdown to steady-state productivity ratio (STSPR) which can easily reach 100%. Moreover, the residual shutdown material, considered as waste, can be dramatically reduced by >80% compared to the current standard shutdown procedures. Given the conflicting nature of the maximization of on-spec production and minimization of the shutdown time, multiobjective optimization of the shutdown operation was also addressed to identify the set of Pareto optimal solutions. Finally, a multicriteria decision-aiding method, based on multiattribute utility theory, was proposed to rank the Pareto optimal solutions to support the decision-making and help identify a suitable and feasible single optimal shutdown solution.

## Introduction

Continuous manufacturing is becoming increasingly popular in the pharmaceutical industry due to its flexibility and significant advantages, such as reduced costs and enhanced productivity and flexibility.^1^ However, the achievement of the full potential of continuous pharmaceutical manufacturing is still hindered by several technical challenges associated with process/plant design, operation, and control.^2^ For example, there is a lack of optimal strategies to operate the plant during dynamic transitions, particularly under short operation windows.^3^ Continuous processes are designed for steady-state manufacturing. Consequently, products generated during startup and shutdown are regarded as wastes.^3,4^ These waste materials produced during the transition phases are complex mixtures with significant amounts of impurities and inconsistent concentrations which make it very challenging to recycle.^1^ In addition, pharmaceutical plants are often built for multiple products, and as a result, the processes are cleaned after each operation to fulfill the regulatory requirements based on which all residual materials are discarded as wastes. The waste generated during the shutdown is more significant in continuous processes involving large inventories^5^ and industries exhibiting shorter operating windows and/and multiproduct manufacturing such as Pharma.^6−8^ This commonly requires multiple cycles of highly regulated cleaning, startup, and shutdown procedures. At the end of a steady-state operation, the continuous plant is commonly shut down with relatively large amounts of residual material.^1,9^ Optimal startup and shutdown strategies may be used to address some of these critical issues to rationalize resource consumption, reduce waste, and improve efficiency. The achievement of this objective requires the development of systematic and rigorous approaches to optimize the dynamic performance of individual and integrated continuous pharmaceutical processes.

Whereas startup optimization aims at a fast attainment of steady state operation,^10^ optimal shutdown procedures may be developed to convert the residual material, at the end of the steady state campaign, to on-spec products under open loop or closed loop dynamic operating conditions. Over the last few decades, the development of optimal startup strategies received more interest compared to the shutdown optimization due to the inherent complexity but also due to a lack of interest in waste minimization and resource efficiency in many sectors. With the current pressure to adopt more sustainable manufacturing and a circular economy, there is a real incentive to minimize waste and achieve high manufacturing efficiency at all development and operation stages. Shutdown optimization studies are relatively scarce and most of the published work to date focused on safety^11,12^ or emergency shutdown,^13^ but only few investigated the maximization of productivity and reduction of wastes. For example, Luperini-Enciso and coauthors proposed closed-loop optimal startup and shutdown strategies for a simulated moving bed chromatography using model predictive control with piece-wise constant profiles.^14^ The objective of the study was to improve the transient performance and maximize product quality. Wood and coauthors proposed a model-based optimal shutdown procedure for a combined cycle gas turbine under limited residual natural gas, to maximize power generation and economic profit.^15^ Another study focused on the Optimal startup and shutdown of a recombinant strain continuously stirred bioreactor,^14^ where the infinite-dimensional dynamic optimization problem was converted into a nonlinear optimization problem by approximating both the model states and control profiles using a family of polynomials defined on finite elements. To minimize the shutdown time, the authors employed a strategy that involved manipulating 20 discretized time intervals for the key parameters, including biomass and substrate concentrations, reactor volume, as well as input and output flow rates. However, to date, no method has been proposed to address the shutdown optimization of a crystallization process.

Crystallization is a key purification technology that is widely adopted in most upstream pharmaceutical processes. The critical quality attributes (CQA) of the drug products, such as tablets and capsules, strongly depend on the critical performance of the crystallization step and its intermediate quality attributes, such as crystal size distribution and purity.^15^ To achieve the targeted quality attributes, it is paramount to implement effective optimization and control strategies in the crystallization processes. Various model-based and model-free techniques have been developed to address optimal operation and control of continuous crystallization processes^16,17^ which focused on three moan classes of processes, which are the mixed suspension mixed product removal (MSMPR) crystallizers,^10^ plug flow crystallizers,^18^ and continuous oscillatory baffled crystallizers.^19^ With model-based approaches, the population balance models were generally considered to capture size and shape distributions over time and enable effective process control or optimization. The optimization strategies were mainly based on the manipulation of a few variables such as cooling temperatures,^20^ antisolvent flow rates,^10^ or through direct control of the supersaturation levels.^21^ Model-free techniques have also been adopted to deliver feedback control strategies that rely on real-time measurements obtained by advanced Process Analytical Technologies (PAT). These measurements deliver direct or indirect information about various CQAs of the product, including the population numbers and size, shape, and polymorphic form distribution. The most popular model-free techniques include direct nucleation control (DNC) and supersaturation control (SSC).^22^

Although continuous crystallizers allow steady-state manufacturing, their overall environmental and economic performance still depends on the effectiveness of the startup and shutdown operations.^23−25^ The development of systematic optimization for startup to minimize the time required to reach a steady state has already been investigated based on different scenarios and using multiple decision variables and optimization strategies. This includes the identification of the discrete jacket temperature profiles, antisolvent flow rates, seeding policies, the initial state of the crystallization vessels, and several discretization methods.^10^ However, despite the significant impact of the shutdown procedure on the cost and environmental performance of continuous pharmaceutical processes, given the large amounts of waste generated at the end of the standard shutdown procedures, the problem has seldom been reported in the literature. Indeed, pharmaceutical manufacturing commonly exhibits high production costs, large environmental footprints, expensive raw materials, and long lead times. The development of effective shutdown strategies is crucial to reduce the overall costs and environmental emissions while improving the resilience and flexibility of the current and future continuous pharmaceutical manufacturing campaigns.

This study proposes the first proof-of-concept of systematic model-based optimal shutdown procedures of a multistage continuous crystallization process. To lay the ground for systematic shutdown strategies of integrated continuous pharmaceutical plants, the focus of the current work is on sequential shutdown strategies of a multistage continuous crystallization process. The approach focused on the maximization of on-spec production and minimization of the shutdown time by using different sets of decision vectors, such as the jacket temperatures and antisolvent flow rates, and adopting various discretization methods. To validate the proposed strategies, a three-stage continuous cooling antisolvent crystallization of aspirin in a mixture of ethanol (solvent) and water (antisolvent) was considered. A set of single optimization scenarios was proposed and discussed to identify the most cost-effective and environmentally friendly shutdown options. Due to the conflicting nature of the maximization of on-spec production and minimization of the shutdown time, a multiobjective optimization of the shutdown operation was proposed to help identify a large set of operating compromises, represented by the nondominated or Pareto optimal solutions. Finally, the Pareto solutions were ranked based on a multicriteria decision-aiding method, which captures the decision-maker preferences alongside the desired tolerance of the objective function with respect to the optima of the single objectives regarded as references. The proposed decision-aiding method allowed the identification of one single shutdown option, which receives the best optimization compromises.

## Material and Methods

**Figure 1:**
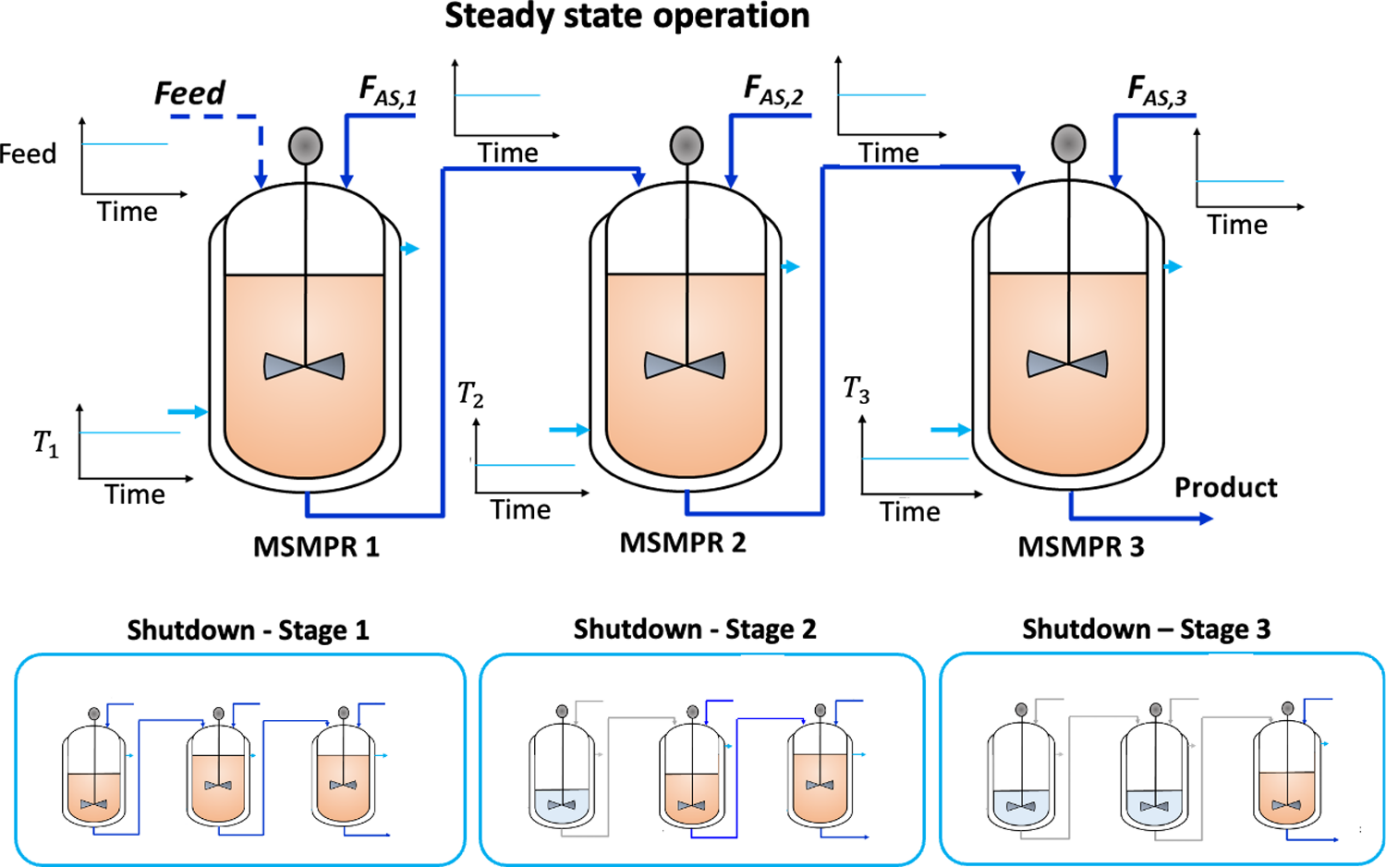
Set-up of the three-stage MSMPR steady-state and shutdown optimal operation.

The crystallization of Aspirin (acetylsalicylic acid, ASA) in ethanol (solvent) and water (antisolvent) is used as a case study to demonstrate and validate the proposed optimal shutdown strategies. A three-stage MSMPR crystallizer was considered, as shown in Figure 1. The objective is to identify the optimal shutdown dynamic operating profiles of the antisolvent flow rates and jacket temperatures that guarantee the production of on-spec products. To explore the most feasible and cost-effective optimal operating strategies during shutdown, the proposed method also investigates the impact of fixed and flexible total shutdown as well as the benefits of several discretization methods. The mathematical model of the multistage MSMPR was adopted from the literature,^10^ based on several key assumptions which include well-mixed vessels, negligible crystal breakage and agglomeration, and total volume is not affected by mixing solvent and antisolvent, crystallization, and dissolution. The mathematical model of the multistage MSMPR was adopted from the literature,^10^ based on several key assumptions which include well-mixed vessels, negligible crystal breakage and agglomeration, and total volume is not affected by mixing solvent and antisolvent, crystallization, and dissolution. In addition, uniform residence time distributions are assumed during shutdown operation. This means that crystal particles and solute have the same residence time at a given vessel, which corresponds to the theoretical mean residence time (i.e., total volume divided by the total outlet flow rate) at any time during shutdown.

When the shutdown begins, the inlet flow rate or fresh feed to the first crystallizer is terminated, and only the antisolvent addition is allowed. Hence, the dynamic population balance model at each stage can be formulated by
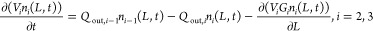
1
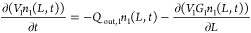
2*n*_*i*_(*L*, *t*) crystal number density (#/cm^3^) at the *i*th stage, and *Q*_out,__i_, *V*_*i*_, and *G*_*i*_ are the outlet flow rate, the total volume of the mixture, and growth rate at the *i*th stage, respectively. The nucleation rate (*B*), crystal growth rate (*G*), solubility (C*, g/kg solvent), and relative supersaturation (*S*) have the same expressions as adopted from the literature.^10,26^
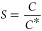
3

The total mass balances expressed in terms of the total volume of material present at the first and *i*th stage (*V*_*i*_) at any time are given by eqs 4 and 5 below.
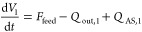
4
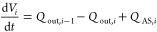
5where *Q*_out,*i*–1_ is the outlet flow rate from the (*i* – 1)th stage, which corresponds to the inlet flow to the *i*th stage, *Q*_out,*i*_ is the outlet flow rate at the *i*th stage, and *Q*_AS,*i*_ is the antisolvent flow rate at the *i*th stage.

It is worth mentioning that the total volume *V*_*i*_ at each stage is expected to decrease during shutdown as opposed to steady state operation, where it is maintained constant at each stage. The shutdown time of the *i*th stage depends on the total net outlet flow rate *Q*_E,*i*_ presented in eq 6 below. *Q*_E,*i*_ may vary during the shutdown, and the total shutdown of a given stage is reached when the vessel is completely empty or when a minimum total volume is reached, which is considered in our case as discussed in the subsequent section.
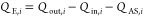
6where *Q*_E,*i*_ is the total net outlet flow rate (total outlet–total inlet).

For the first crystallization stage, the fresh feed (*Q*_in,1_) is terminated at the start of the shutdown operation. Similarly, the inlet flow rate (*Q*_in,*i*_) at the *i*th stage is terminated when the previous crystallization stage reaches the final shutdown condition (i.e., when it is completely empty or when a minimum total volume is reached).

The standard method of moments is used to solve the population-balanced model described by eqs 7 and 8. With the standard method of moments, the population-balanced model under variable total volume is converted to the set of ordinary differential equations described below.
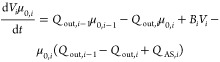
7
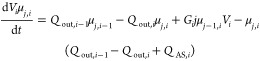
8

where *B*_*i*_ and *G*_*i*_ represent the nucleation and growth kinetics at the *i*th stage, respectively,^25^ and μ_*j*,*i*_ is the *j*_th_ moment at the *i*th stage for the characteristic dimension *L*_*i*_ is described by
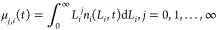
9

At the beginning of the shutdown operation, the fresh feed to the first crystallization stage is switched off. Consequently, the moment equations can be written as
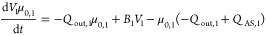
10
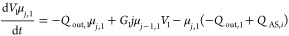
11

Similarity, eqs 7 and 8 above can also be simplified in the case of crystallizers 2 and 3 when the inlet flow rates are terminated.

The components mass balances at each stage can be described as follows:
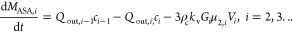
12
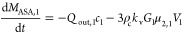
13
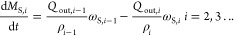
14
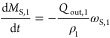
15
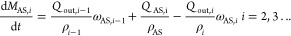
16
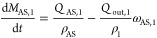
17where ω_AS,*i*_(*t*) is the antisolvent ratio in the solvent mixture, *M*_*i*_ is the mass of API, solvent, and antisolvent on the *i*th stage. The *c*_*i*_ is the concentration of API on the *i*th stage and ρ_c_ is the density of the crystal.

The energy balance equations were adopted from the literature.^10^*M*_OS_ is the total mass of the on-spec products obtained during the shutdown operation. The crystal products are considered on spec if the critical quality attribute, here the mean crystal size, is within ±5% of the targeted steady state value (417 μm). The total on-spec production can be calculated by
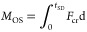
18where the *F*_cr_ is the mass flow rate of on-spec crystals and *t*_SD_ is the moment in time when the crystal properties no longer fall within the specified quality requirement range. More CQA to the problem is certainly feasible and will result in more constraints, which may make the optimization more challenging from a feasibility perspective.

Based on the assumption of well-mixed vessels, the mass flow rate of on spec crystals can be obtained by
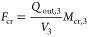
19where *M*_cr,3_ is the total crystal mass in the third stage, which can be calculated by
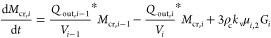
20
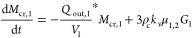
21

The environmental factor (E-factor), which is presented in eq 22, can be used to capture the environmental footprint. This may be considered as one of the key performance indicators in the subsequent section or used as one of the objective functions in the optimization problem.
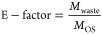
22

To optimize the intended shutdown operation using the model equations above, it is necessary to provide the initial conditions, which represent, in this case, the steady-state conditions. The Supporting Information Table S1 summarizes all key inputs and parameters associated with the mathematical models for each of the crystallization stages. This includes temperature, antisolvent flow rate, mass, concentration, volume, crystal size, and the moments associated with population balance models. These data capture the steady-state conditions, which essentially represent the initial conditions of the model-based optimal shutdown strategy.

### Single Objective Shutdown Optimization

The proposed shutdown procedure follows a sequential approach. When the shutdown operation starts, the fresh feed or inlet flow rate to the first crystallization stage is stopped. As a result, the steady-state conditions (e.g., individual jacket temperatures, antisolvent flow rate, and target volumes), which are associated with single operating points in the phase diagram, become insufficient to guarantee on-spec production, due to the rapid depleting supersaturation. When the feed is switched off at the start of the shutdown stage, the only source of additional solute (active pharmaceutical ingredient, API) for crystallization is removed. As a result of crystal growth and eventually nucleation, supersaturation inevitably starts to drop if the process is maintained at the steady state conditions of temperature and antisolvent ratio. The supersaturation will drop until equilibrium conditions are reached. In essence, if steady-state conditions (i.e., constant temperature and constant antisolvent flow rate at each stage) are used during the shutdown, all obtained crystals will be off specifications due to the poor control of supersaturation. Instead, optimal dynamic profiles of antisolvent either the jacket templates, antisolvent flow rates, or both must be identified. These dynamic profiles are aimed to generate enough instantaneous supersaturation to compensate for the absence of the fresh feed while allowing further recovery of the solute or API. Consequently, the optimal shutdown operation of each stage must follow an optimal dynamic trajectory in the phase diagram instead of a single operating point. Based on the proposed sequential shutdown procedure, the second crystallization stage continues to receive an inlet flow rate until the first crystallization stage is fully shut down, in which case, the inlet flow rate is stopped. The same procedure is repeated for the third crystallization stage.

Although it is theoretically possible to recover all or most of the crystals present in the last crystallization stage as on-spec material, assuming they can be recovered, washed, and filtered fast enough immediately after shutdown before deviating from specification due to the presence of supersaturation, the recovery of on-spec material from the first and second stages cannot be achieved without implementing optimal operating profiles. Most importantly, from a general perspective, the final product is obtained at the very last process; as a result, the last stage cannot be stopped or shut down while upstream processes are still operating because the final operating step that delivers the final product is no longer available. These observations suggest that the shutdown procedure of multistage and integrated processes must follow a sequential procedure like the one adopted during startup.^1,3,6^

Several optimization strategies can be investigated to address the proposed optimal shutdown problem of the three-stage MSMPR crystallizer aimed at the maximization of on-spec production. This includes the type and number of the decision variables, the discretization strategy, and fixed vs flexible total shutdown time. Consequently, several single objective optimization scenarios were formulated to maximize on-spec production, as summarized in Table 1. To solve the proposed optimization problem, a control vector parametrization (CVP) approach is based on the discretization of the control vectors into piece-wise constant profiles for the antisolvent flow rates and piece-wise linear profiles with continuities for the jacket temperatures. For the sake of brevity, only a generic shutdown optimization problem is presented in eq 23.
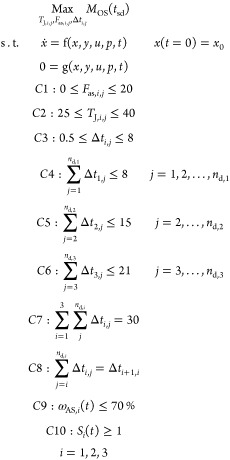
23where *n*_d,*i*_ is the number of discretization intervals of the *i*th stage. *F*_as,*i*,*j*_ is the antisolvent mass flow rate (g/min) added to the *i*th stage during *j*th time interval (min), and *T*_J,*i*,*j*_ is the jacket temperature (°C) of the *i*th stage at the end time point of *j*th time interval.

**Table 1:** Summary of the Settings for the Proposed Shutdown Optimization Scenarios

shutdown scenario	decision vectors/variables	discretization time intervals	discretization time intervals	total shutdown time
scenario 1	*T*_J,1,*j*_	crystallizer 1:5 intervals	5 equal intervals	
	*T*_J,2,*j*_	crystallizer 2:6 intervals	one fixed and 5 equal intervals	30
	*T*_J,3,*j*_	crystallizer 3:7 intervals	2 fixed and 5 equal intervals	
scenario 2	*F*_as,1,*j*_	crystallizer 1:5 intervals	5 equal intervals	
	*F*_as,2,*j*_	crystallizer 2:6 intervals	one fixed and 5 equal intervals	30
	*F*_as,3,*j*_	crystallizer 3:7 intervals	2 fixed and 5 equal intervals	
scenario 3	*T*_J,1,*j*_,*F*_as,1,*j*_	crystallizer 1:5 intervals	5 equal intervals	
	*T*_J,2,*j*_,*F*_as,2,*j*_ *T*_J,3,*j*_	crystallizer 2:6 intervals	one fixed and 5 equal intervals	30
	*F*_as,3,*j*_	crystallizer 3:7 intervals	2 fixed and 5 equal intervals	
scenario 4	*T*_J,*i*,*j*_	crystallizer 1:5 intervals		
	*F*_as,*i*,*j*_	crystallizer 2:6 intervals	flexible	
	Δ*t*_*i*,*j*_	crystallizer 3:7 intervals		
scenario 5	*T*_J,*i*,*j*_	crystallizer 1:5 intervals		
	*F*_as,*i*,*j*_	crystallizer 2:10 intervals	flexible	
	Δ*t*_*i*,*j*_	crystallizer 3:15 intervals		
scenario 6	*T*_J,1,*j*_,*F*_as,1,*j*_	crystallizer 1:5 intervals	5 equal intervals	
	*T*_J,2,*j*_,*F*_as,2,*j*_*T*_J,3,*j*_	crystallizer 2:6 intervals	one fixed and 5 equal intervals	30
	*F*_as,3,*j*_	crystallizer 3:7 intervals	2 fixed and 5 equal intervals	

In the mathematical formulation of the generic optimization problem, the constraints *C*1–*C*3 capture the lower and upper bounds of the decision variables, *C*4 to *C*6 represent the maximum shutdown time allowed for the *i*th stage (expressed in minutes), *C*7 sets an upper limit to the total shutdown time of all crystallizers (expressed in minutes), *C*9 helps maintain the antisolvent ratio below 70% at all stages at any time, and *C*10 helps maintain a minimum, relative supersaturation (S) of 1 to avoid dissolution during shutdown.

Based on the optimization problem and assumptions above, several optimization scenarios were considered (Table 1) that correspond to different mathematical formulations. The discretized jacket temperature profiles of the *i*th stage and time intervals were considered as the decision vectors in Scenario 1 (constraints used: *C*2, 7, 8, and 10), and the antisolvent flow rates were considered as decision variables in Scenario 2 (constraints used: *C*17, 8, 9 and 10). The combined optimization of jacket temperatures and antisolvent flow rates is considered in Scenario 3 (constraints used: *C*1, 2, 7, 8, 9, and 10). In the three first scenarios, the shutdown time was fixed as 30 min. The idea is to maximize the recovery of on-spec crystals under fixed shutdown time. The discretization method is also a critical optimization parameter, and as such, Scenario 4 was developed based on the combination of several decision vectors including jacket temperatures, antisolvent flow rates, and time intervals (constraints used: *C*1–10). In Scenario 4, the shutdown time of each stage is not fixed.

With the first single optimization problems in Scenarios 1–3, 5 discretization intervals were proposed for the first stage, 6 for the second stage, and 7 for the third stage. Based on this approach with the proposed 6 discretization intervals for the second crystallization stage, the first time interval, associated with stage 2, is equal to the total shutdown time of stage one. The same approach is adopted for stage 3 which involves 7 intervals where the 2 first intervals are equal to the total shutdown time of the first stage and second stage receptively, as described mathematically by the equality constraint *C*8. This approach helps reduce the computational burden by reducing the number of required decision variables. As a result, while the optimizer is identifying the optimal piece-wise constant of the antisolvent flow rates and piece-wise continuous profiles of the temperature (5 intervals) for the first crystallization stage, it identifies one single constant flow rate and one cooling/heating trajectory during the first discretization time interval of the second shutdown stage (second crystallizer) followed by the optimal five-interval based piece-wise constant and piece-wise continuous profiles. The same approach applies to the third stage which has two first large intervals, with constant flow rates and fixed cooling/heating rates, followed by the optimal 5-interval-based piece-wise constant and piece-wise continuous profiles.

In scenarios 1 to 3, the target shutdown time was fixed at 30 min, and each stage was shut down sequentially. Scenarios 1 to 3 were designed to investigate the impact of optimal cooling profiles, antisolvent additions, and their combination based on constant discretization schemes.

Scenarios 4 and 5 were proposed to investigate more advanced and computationally costly options based on more complex discretization strategies. As such, the proposed scenarios involve the optimization of the discretization time intervals, which are no longer considered equally spaced. In addition, the shutdown time is no longer fixed to explore possible ways to improve the overall shutdown performance. The objective is to investigate the impact of the proposed flexible time intervals and identify the overall best optimal shutdown compromises.

## Results and Discussion

The proposed optimal control problems introduced earlier were solved using the CVP method, one of the most popular methods, which converts the infinite-dimensional problem into a discrete problem that can be solved using nonlinear optimization solvers.^26^ Other methods can also be used, which include single or multiple shooting or collocation methods (i.e., state and control parametrization). With the proposed CVP in the presented work, a hybrid nonlinear optimization method, which combines a genetic algorithm and a local gradient-based optimizer, was developed in MATLAB. First, the problem is solved with a genetic algorithm to deliver a good estimate of the global solution. The solution is then fed to the local gradient-based optimizer (fmincon) for further refinement. The initial conditions for the differential-algebraic system involved the optimization problems, formulated in eq 25, which correspond to the steady-state values summarized in Table S1.

**Figure 2:**
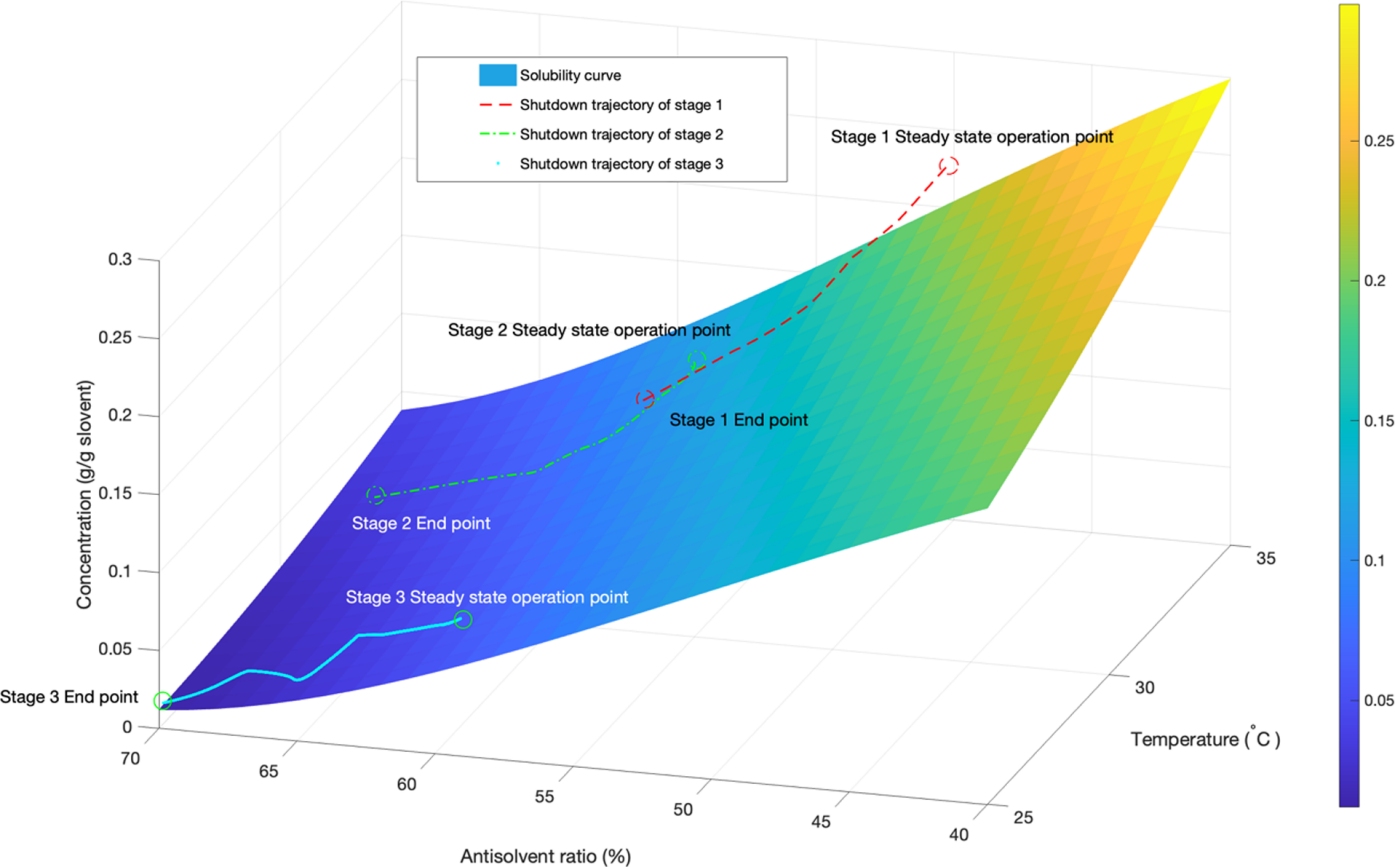
Optimal shutdown trajectories in the phase diagram.

The first two optimal shutdown scenarios are based on the manipulation of the operation profile of the jacket temperatures (Scenario 1) and the antisolvent flow rates (Scenario 2). Figure 2 shows the optimal jacket temperature profiles and the corresponding dynamic profile of the total solvent and antisolvent mass at each stage and on spec production.

The shutdown of each stage is assumed to be completed when the total mass at a given crystallization stage reaches 20% of the total mass at the steady state. A lower mass than 20% is not considered due to poor or no mixing for such a small hold-up volume. For the very last crystallization stage (here the third stage), which determines the point at which shutdown is complete, the terminal condition is met when either the volume reaches a minimum capacity (i.e., 20%), a fixed total shutdown time is reached, or crystal products become off spec when the total shutdown is not fixed.

**Figure 3:**
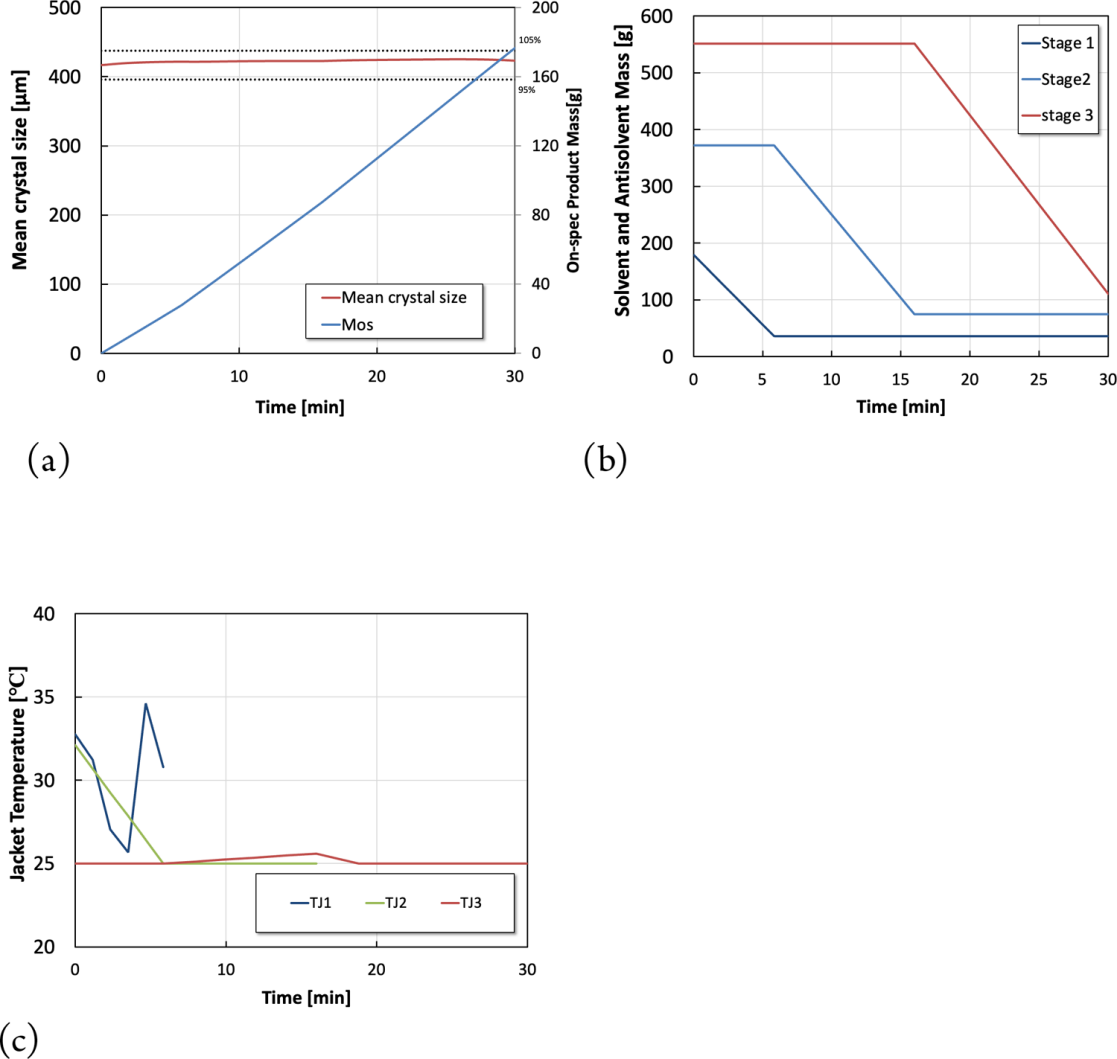
Optimization results for scenario 1. (a) Dynamic profile of the mean crystal size and total on spec production. (b) Total solvent and antisolvent mass at each stage and total on spec product. (c) Optimal jacket temperature profiles.

Based on the optimal operating temperature profiles obtained for Scenario 1, the predicted total on-spec production is 177.6 g. Figure 3a confirms that the mean crystal size is maintained within the targeted quality band or specifications (±5%) until the end of the shutdown operation, after 30 min. Figure 3b shows how the continuous crystallizers are shut down sequentially, which happens when the total volume at a given stage reaches 20% of the steady-state total volume. In Figure 3c, the jacket temperature at stage 1 experienced substantial changes to compensate for the depleting supersaturation due to the absence of fresh feed. Similar temperature patterns can be observed in crystallization stages 2 and 3 when the inlet flow rates are switched off. Furthermore, as expected, the jacket temperature in stage 2 is kept at an optimized operation state during the first time interval, which corresponds to the total shutdown of stage 1 (Figure 3c), and similarly, the jacket temperature in stage 3 is kept at an optimized operation state during the first and second time intervals, which correspond to the total shutdown of stages 1 and 2.

**Figure 4:**
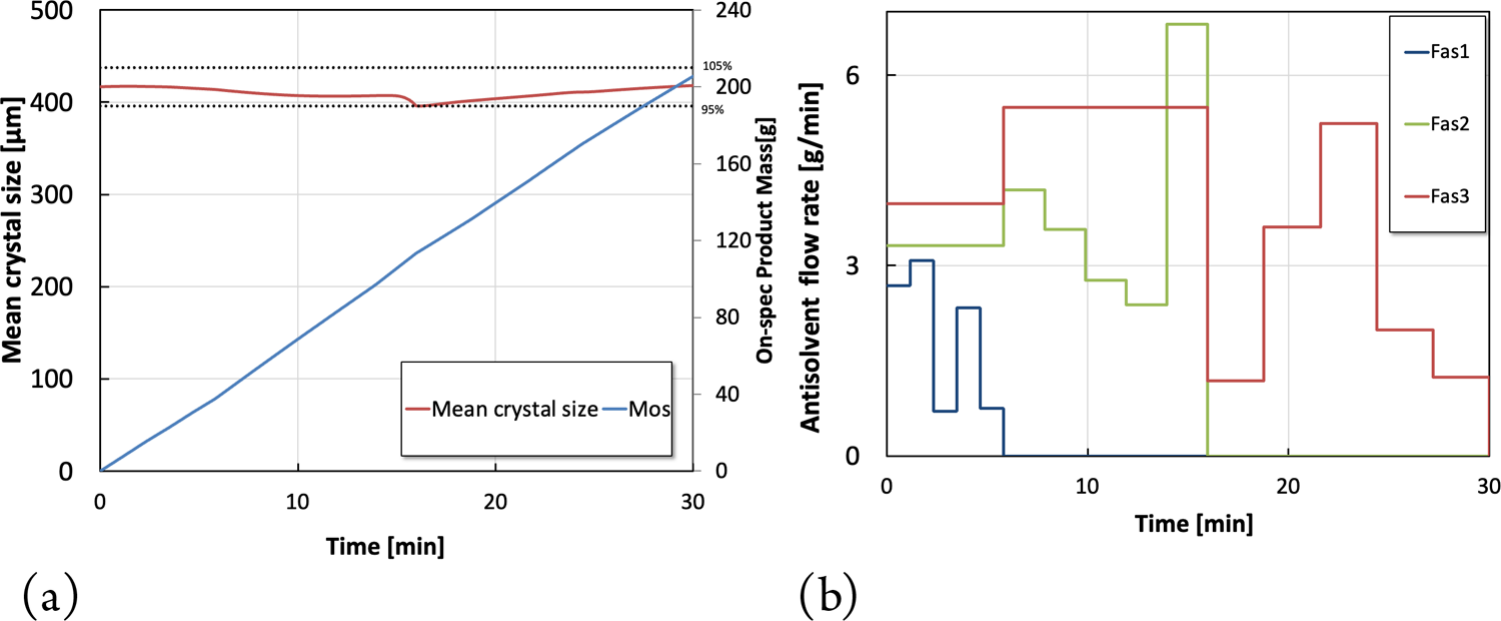
Optimization results for scenario 2. (a) Dynamic profile of the mean crystal size and total on spec production. (b) Optimal antisolvent piece-wise constant profiles.

In Scenario 2, only the antisolvent flow rates at each stage are used as the decision variables, with all jacket temperatures being set at their steady-state values. The results associated with Scenario 2 are shown in Figure 4.

The optimal antisolvent flow rate profiles shown in Figure 4b correspond to a total on-spec production of 205.6 g during shutdown based on Scenario 2, where the jacket temperatures are kept constant at their steady-state values. Figure 4a shows that the mean crystal size is indeed maintained within an acceptable range during shutdown. The shutdown process was divided into three stages, with the antisolvent flow rate of the second and third stages maintained constant during the shutdown of the first stage, which was also regarded as a decision variable in the optimization problem. It is worth noting that the corresponding constant antisolvent flow rate values were optimized, rather than being set to their steady-state values.

In scenario 2, the antisolvent flow rates in the first stage and total antisolvent added were lower than those in subsequent stages due to the smaller steady-state volume of material in stage 1, besides the fact that the antisolvent to solvent ratio must be below 70% (constraint *C*7). During the shutdown of the first crystallization stage, the antisolvent flow rates into the second and third stages were maintained at optimal values determined by the optimizer. Once the optimal shutdown of the second stage is initiated, the antisolvent flow rate becomes an optimized piecewise constant profile, while the antisolvent flow rate into the third stage remains constant. This optimization procedure was adopted to minimize the computational cost.

**Figure 5:**
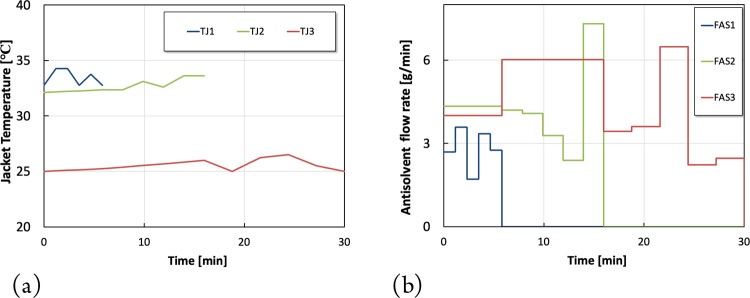
Optimization results for scenario 3. (a) Optimal jacket temperature piece-wise continuous profiles. (b) Optimal antisolvent piece-wise constant profiles.

In scenario 3, where both antisolvent flow rates and jacket temperatures are used as the decision vectors, the optimal shutdown policies result in a predicted on-spec production of 208 g. Figure 5a shows that the optimal jacket temperature profiles are significantly different from those observed in Scenario 1. In Scenario 1, the jacket temperatures provide the only way to manipulate supersaturation, which is the driving force of crystallization, whereas in Scenario 3 both jacket temperatures and antisolvent flow rates were used to manipulate the supersaturation and maximize the targeted on-spec production. This explains the resulting smooth temperature profiles (low cooling/heating rates) depicted in Figure 5a compared to the sharper temperature profiles in scenario 1 (Figure 3) where more aggressive temperature changes are required to cause sufficient/significant effect on supersaturation. It becomes clear that the addition of antisolvent in scenario 3 provides more flexibility and enhanced degrees of freedom to achieve the targeted supersaturation levels, thereby reducing the need for significant changes in the jacket temperatures.

In addition to the decision variables discussed above in Scenarios 1 to 3, the shutdown time can also be considered as a decision variable, as discussed in Scenarios 4 and 5. It is worth noting that the discretization time intervals for each crystallization stage were fixed in the first scenarios to simplify the optimization problem. Scenarios 4 and 5 were developed precisely to investigate the impact of variable/optimized discretization time intervals on optimal shutdown performance.

Given the significant difference in the total volume present at each crystallization stage and the critical industrial requirement to achieve a complete shutdown within a reasonable time, three additional constraints *C*4, *C*5, and *C*6 were considered, as described in eq 23.

**Figure 6:**
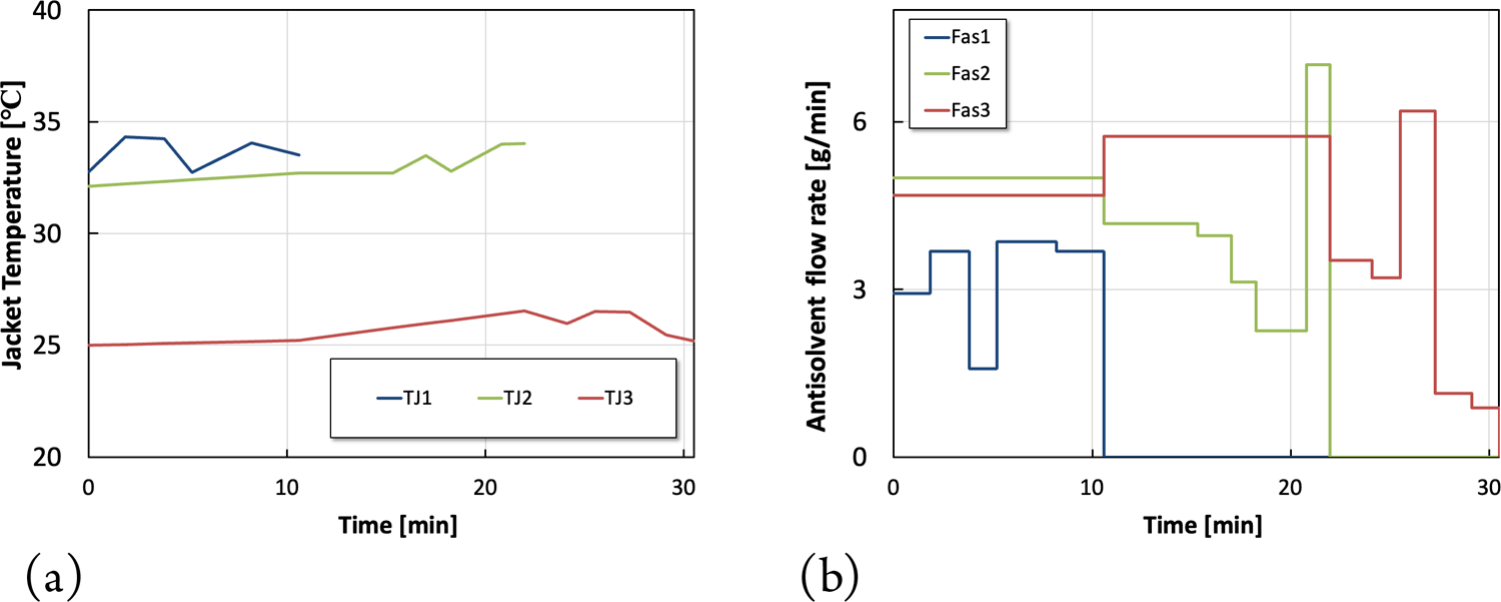
Optimization results for scenario 4. (a) Optimal jacket temperature piece-wise continuous profiles. (b) Optimal antisolvent piece-wise constant profiles.

The optimization results associated with scenario 4 are listed in Figure 6. These results show that allowing the total shutdown time to change within a reasonable range may prove very beneficial. Indeed, Scenario 4 shows that an optimal total shutdown time of 30.52 min, which is slightly longer than the previously fixed shutdown time of 30 min in scenarios 1–3, results in a total on-spec production of 217.8 g of on-spec products which compared to scenario 1 represents an increase of productivity of 22.6%. Figure 6a shows a slight increase in the jacket temperatures. During the shutdown process, the fresh feed, which is at a higher temperature, is no longer added and only antisolvent is continuously added at a much lower flow rate. This may explain the slight increase in the optimal jacket temperatures compared to Scenarios 1 and 3 as shown in Figure 6. These temperature changes are required to meet the constraints (*C*8) by preventing large supersaturation levels that could lead to significant nucleation and, consequently, large numbers of crystal fines which may result in a significant deviation from the targeted quality specifications.

The additional scenario 5 was developed to investigate another optimization option where the jacket temperatures and antisolvent flow rates of the second and third stages are no longer maintained constant during the shutdown of the previous stages but are also manipulated based on the proposed discretized profiles. The proposed scenario is meant to determine whether the simplified optimal shutdown strategies discussed earlier in Scenarios 1–3 can be outperformed to help identify the best shutdown compromises by evaluating both complexity and computational costs. Compared to Scenario 4, Scenario 5 uses a larger number of decision variables, as the number of discretization intervals is equal to 5 for the first stage, 10 for the second stage, and 15 for the third stage. In summary, this means that the antisolvent flow rates and jacket temperatures associated with the second and third crystallization stages are simultaneously optimized along the first stage based on the same discretization schemes, and the same holds for the last crystallization stage when the second is being shut down.

**Figure 7:**
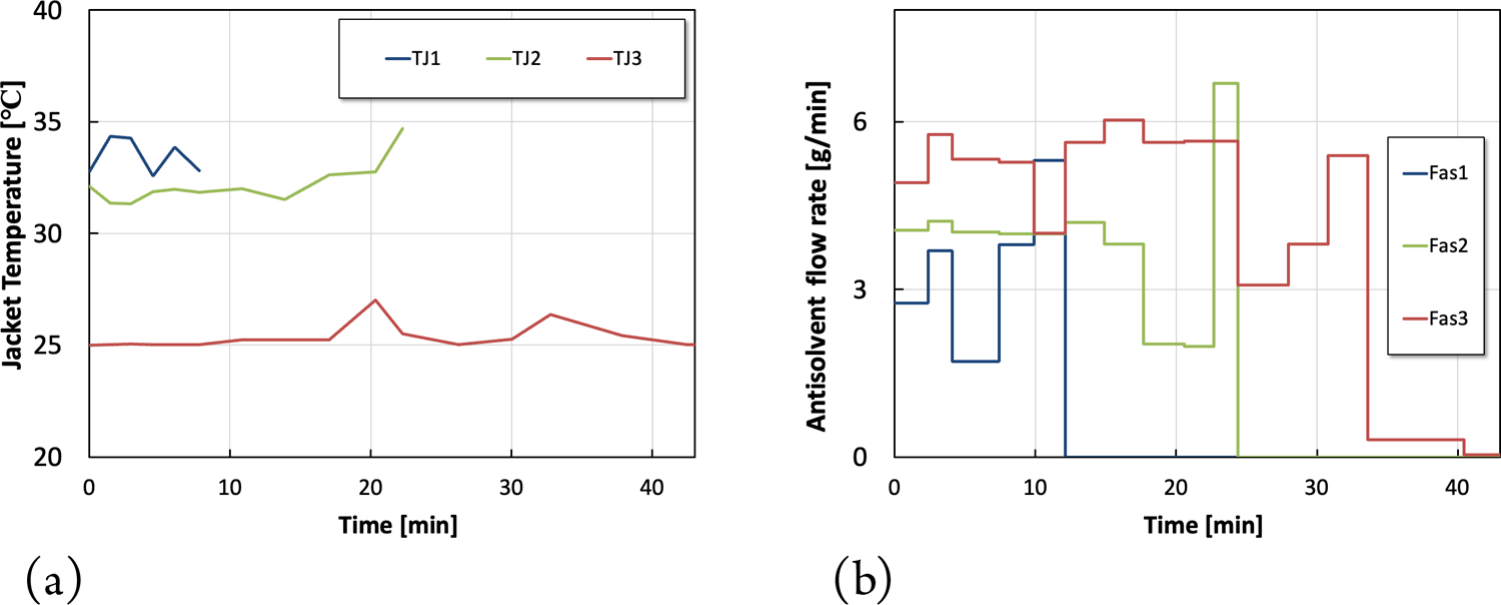
Optimization results for scenario 5. (a) Optimal jacket temperature piece-wise continuous profiles. (b) Optimal antisolvent piece-wise constant profiles.

The optimization results associated with Scenario 5 are presented in Figure 7. The optimal shutdown procedure in Scenario 5 resulted in a maximum on-spec production of 222 g, which represents a slight improvement of 5 g compared to Scenario 4. However, the corresponding shutdown time was increased to 48 min. Besides, Scenario 5 requires 12 additional decision variables that dramatically increase the computational cost. As a result, the discretization strategies used in Scenarios 1- 4 are overall reasonable and deliver good compromise despite the slight improvements associated with Scenario 5.

The first 5 scenarios provide different options for the intended sequential shutdown strategy, laying the ground for more systematic optimal shutdown procedures of more complex continuous processes/plants which may integrate several continuous operating steps (e.g., reaction, filtration, drying). The shutdown is conducted sequentially to maintain a constant feed to the subsequent or downstream processes such as filtration drying, granulation, etc. An additional Scenario 6 was developed based on the settings adopted in Scenario 3 under the ideal assumption based on which all material present in each continuous crystallizer can be fully pumped out during the shutdown procedure. This suggests no loss of mixing efficiency while the vessel is being fully drained, and as a result, no residue is left in the vessel at the end of the shutdown. If this assumption is technically possible, Scenario 6 may become an advantageous option given that more on-spec products can be collected while the residues are being minimized.

**Figure 8:**
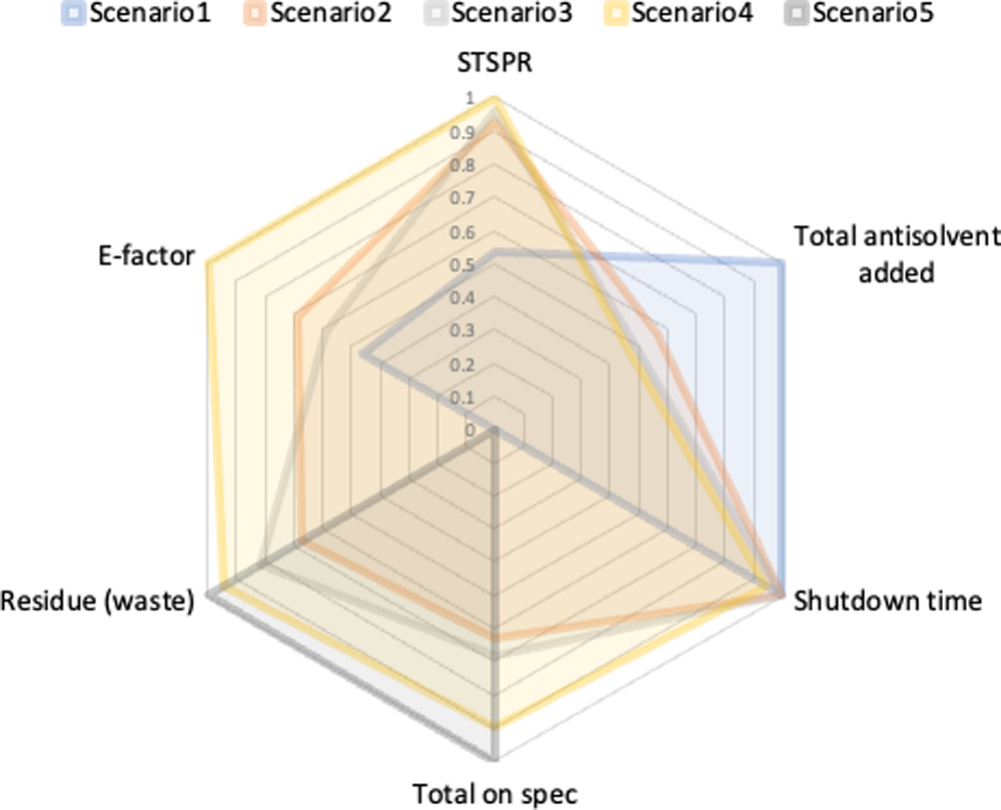
Radar chart of the KPI for the different scenarios.

Table 2 summarizes the key performance indicators (KPI) associated with the 6 scenarios described above, which provide more insights and lay the ground for more holistic decision-making. First, a new critical performance indicator was introduced to capture the shutdown efficiency based on the STSPR. The latter can be calculated by taking the ratio between the total on-spec production, for a given shutdown time, and the total (on-spec) steady-stateon-spec production for the same duration. In essence, the proposed metric provides a critical insight into the general cost vs time efficiency of the shutdown procedure. Table 2 also summarizes the performance of each scenario based on the total on spec production, the environmental footprint, which is captured based on the E-factor (i.e., the ratio between the total mass wastes and total mass of on-spec products), the total amount of antisolvent used during the shutdown, as well as the total residual material left in the vessels at the end of the shutdown. To allow a more effective and clear comparison between the different scenarios, the six KPIs are compared using the radar chart presented in Figure 8. Radar charts are commonly used to assess and analyze multiple criteria at the same time. Here, the objective is to build a holistic view that summarizes the pros and cons of each of the proposed scenarios. Due to the conflicting nature of the proposed KPI, where a few of them are required to be maximized, whereas the others are required to be minimized, it is important to introduce a normalization approach. Consequently, the normalized distance between the current value and the optimum is used, as described by the equations below. We can either convert all KPI scores to a maximization and identify the scenario that displays the largest surface area in the radar chart or convert all KPI scores to a minimization and identify the scenario with the smallest surface area. Here, the KPI scores were normalized according to eqs 24 and 25 below using the normalized distance from the maximum, for KPI that require minimization (eq 24), and distance from the minimum for KPI that require maximization (eq 25). The difference between the maximum and minimum scores is used as the normalization term in the denominator. In summary, eq 25 was used for the total on-spec production and STSPR, whereas eq 24 was used for the shutdown time, total antisolvent added, residual material, and E-factor.
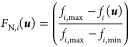
24
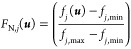
25where *F*_N,*i*_ is the normalized KPI.

**Table 2:** Summary of Key Performance Indicators Associated with the Proposed Single Objective Sequential Shutdown Optimization Scenarios

scenarios	number of decision variables	shutdown time (min)	total antisolvent added (g)	residual material (g)	E-factor	total on spec production (g)	STSPR
1	18	30	0	283.8	6.90	177.6	0.87
2	18	30	186.5	269.7	6.73	205.6	1.01
3	36	30	220.3	266.8	6.80	208.0	1.02
4	51	31	229.6	264.1	6.49	217.8	1.03
5	75	48	431.3	262.9	7.25	222.3	0.68
6	36	30	218.73	0	5.61	245.4	1.20

By comparing the KPI of all optimal shutdown scenarios in Table 2, which are captured more clearly in the radar chart in Figure 8, Scenario 6 delivers the best performance as clearly shown by the largest on-spec production, lowest E-factor, and the low volume of antisolvent required. Besides, it delivers a higher productivity rate compared with steady-state operation. However, this scenario was designed under ideal conditions, and it may not be realistic or feasible due to technical issues such as the loss of mixing efficiency during shutdown, as discussed previously. The next most interesting scenario seems to be Scenario 5 which delivers the largest total on spec production. However, this scenario exhibits the largest shutdown time along with the most complex and computationally costly alternative. Besides, the total volume of antisolvent added in Scenario 5 was dramatically increased, which may significantly increase the operating costs. The antisolvent used in this case is water, which is relatively readily available. However, water is seldom used in pharmaceutical manufacturing, and most reaction and crystallization processes use organic solvents; besides, the pharmaceutical sector is the most solvent-intensive industry. Moreover, wastes, that contain water alongside Aspirin, ethanol, and impurities, require further purification, which comes with additional operating costs and environmental impact. These will result in an increased total operating cost. Consequently, Scenario 4 may represent the best compromise between complexity and required degrees of freedom to deliver increased productivity, despite the slightly larger shutdown time compared to Scenario 1- 3. Scenario 3 or 2 may also be regarded as a good alternative, given the relatively high productivity and low complexity and computational costs.

After evaluating all sequential shutdown scenarios outlined in Table 2, it becomes apparent that Scenario 4, which was designed under nonfixed total shutdown time, delivers the best compromise between the different KPIs, and it is based on more realistic assumptions compared to Scenario 6. Indeed, Scenario 4 delivers high productivity and low environmental impact under a reasonable shutdown time of 31 min. It is also important to note that in a fixed total operating window of a continuous manufacturing campaign, a longer shutdown time may come at the expense of a shorter steady state operating, which may result in decreased productivity particularly when the STSPR is lower the 100%. Therefore, the development of an effective optimal shutdown strategy may be regarded as a compromise between a shorter shutdown and increased spec production. This suggests that the optimal shutdown strategy may be addressed as a multiobjective optimization (MOO) problem where the shutdown time becomes another objective function as designed in Scenario 4. This approach is the scope of the next section.

The E-factor evaluated based on the steady-state operating conditions is 6.25 by excluding the residual materials at the end of the continuous manufacturing campaign. This suggests that the E-factor values associated with each of the proposed shutdown scenarios deliver similar values that are essentially below 7. This highlights that the optimal shutdown scenarios deliver effective green performance and help convert the residual materials at no extra environmental burden compared to the (theoretical) steady-state E-factor target.

Additional shutdown strategies may be considered if the forward sequential approach is no longer a critical requirement. This is particularly relevant if either the multistage crystallization is a standalone process, the manufacturing plant is a hybrid involving batch and continuous processes, or there is a buffer tank where the crystals can be collected prior to the downstream processing. To explore this avenue, two additional scenarios were investigated as detailed in the Supporting Information. In Scenario 7, the three crystallization stages are treated or optimized separately as semibatch systems during the shutdown. It is worth mentioning that this scenario implies that on-spec crystals will be available at each separate stage only at the end of the shutdown procedure, which suggests that these units will be fully disconnected from the downstream processes. Consequently, all crystallization stages can be optimized independently based on a similar dynamic optimization or optimal control strategy as suggested in the Supporting Information eq S1. Scenario 8 is proposed to capture a reverse sequential shutdown strategy. Here, the multistage crystallizer was shut down, starting from the third to the first stage. However, this scenario implies that the three crystallization stages will be disconnected and optimized separately, while the required on-spec feed to subsequent processing steps is maintained. It is worth emphasizing that although the third crystallization stage contains on-spec crystals, at the beginning of the shutdown procedure, their properties can quickly deviate from the targeted specifications if not recovered instantly due to the outstanding supersaturation since a continuous crystallizer operates at a steady state at a point above the solubility curve.

In summary, the additional Scenario 7 makes it possible to produce 243.84 g of total on-spec material during shutdown, which is slightly lower than Scenario 6. This is because both semibatch and continuous cannot fully use the supersaturation within shutdown time. Both did not reach equilibrium. The overall performance in Scenario 7 may also depend on the available shutdown time, used here as the batch time, but also on the flexibility of the optimization and discretization scheme. Overall, Scenarios 6 and 7 exhibit similar environmental performance, but Scenario 6 requires more antisolvent. Finally, Scenario 8, which is based on a reverse shutdown strategy as detailed in the Supporting Information, exhibits average overall performance compared to the forward sequential shutdown strategies.

### Multiobjective Optimization

It was clearly shown that a shutdown optimization problem may be addressed in several ways based on the available degrees of freedom, computation power, available resources, and targeted maximum shutdown time. This outlines the competing or conflicting nature of these different criteria, which may require a more in-depth analysis to help identify a set of reliable scenarios for the decision-maker. After the identification of the best single objective optimization scenario, a multiobjective optimization strategy can be adopted to deliver a boarder set of compromises or trade-offs to the decision maker/engineers.

Therefore, a new multiobjective optimization problem was set based on the simultaneous minimization of the shutdown time and the maximization of on-spec production based on the settings used in Scenario 4, which exhibited the best overall performance in the single objective optimization problems discussed above. The corresponding MOO problem can be mathematically formulated as shown in eq 26.

Here, the decision variables are jacket temperature, antisolvent flow rate, and discretization time interval length which are discretized using the same approach as in single objective optimization in Scenario 4.

**Figure 9:**
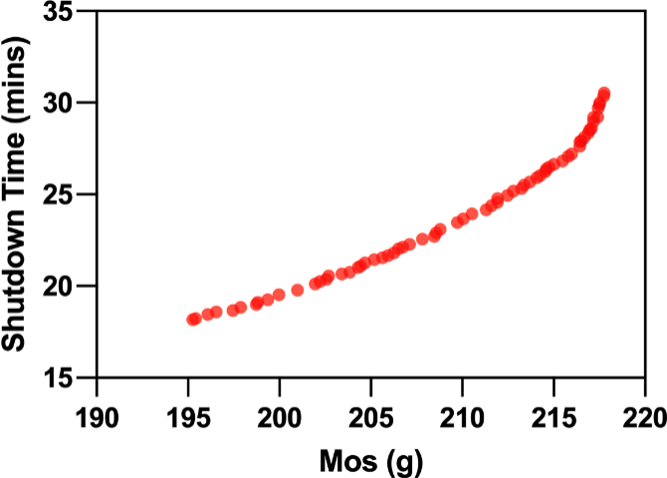
Pareto front representing the minimization of the shutdown time and the maximization of total on spec production.

The MOO problem was solved using a genetic algorithm (gamulti in MATLAB) and the solutions obtained are represented by the Pareto front shown in Figure 9.
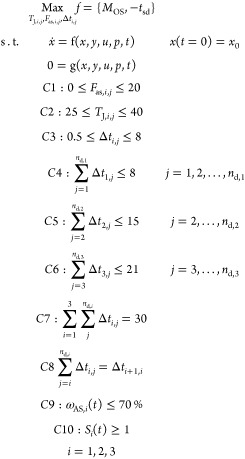
26

Overall, the Pareto front is smooth and consistent with a typical Max Min MOOP problem. The Pareto also shows that the possible ranges for shutdown time and total on-spec production are, respectively, 195–218 g and 18–31 min. Overall, longer shutdown times deliver more on-spec production. A maximum of 218 g on spec production can be achieved within a 31 min shutdown time, and 195 g on spec production can be obtained based on the shortest shutdown time of 18 min.

The multiobjective optimizer delivers a set of Pareto values that provides a set of possible compromises that satisfy the problem constraints. When it comes to implementing one optimal shutdown solution, it becomes critical to enable the operator to implement or test one or just a few possible solutions. This decision-making often requires ranking the Pareto solutions based on additional decision-making criteria. To address this problem, a systematic strategy based on multicriteria decision aiding is proposed to rank the Pareto solutions. More specifically, the multiattribute utility theory (MAUT) is used to capture more effectively the underlying decision preferences. The method allows for a more effective analysis of the decision options to help achieve more reliable and robust decisions. As such, it has been successfully implemented to address several scientific and engineering problems involving MOO.^27^

The MAUT method follows the following key steps:

- Setting an objective and establishing the attributes for the goal.
- Identify a range for the attributes.
- Derive the single utility functions for each attribute.
- Estimating the weighting factors for each attribute.
- Deriving the multiattribute utility function

The single utility functions are used to evaluate the performance criteria individually in the same units.

According to the suggested decision-making preferences, the normalized single utility functions associated with the MOO criteria or objective functions are captured in eqs 27 and 28. The first utility function is chosen to be an increasing function while the second is a decreasing function.
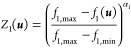
27
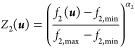
28where *f*_max_ and *f*_min_ are the maximum and minimum values of the objective functions from the Pareto front and α_*i*_ is a utility parameter that impacts the relative tolerance of an objective function.

**Figure 10:**
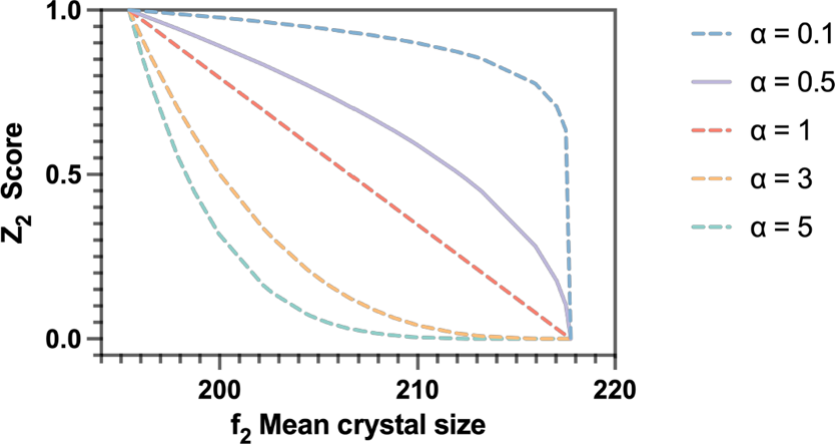
Illustration of the effect of α_2_ on the individual utility function of *Z*_2_.

By evaluating the ratio between the distance from the targeted optima of the single objective functions in the Pareto front, *f*_1,max_ for the function being minimized and *f*_2,min_ for the function being maximized, with respect to the difference between the maximum and the minimum possible values, the utility functions become normalized, which helps establish a more reliable way to compare the performance of a given solution with respect to the best performance of each objective function in the Pareto front. The parameter α_*i*_ is a tuning parameter that captures the relative tolerance by setting a score according to the distance from the targeted optimal solution of a single objective function. For example, if α_1_ is chosen to be large, a solution far away from the targeted best performance (*f*_1,max_) will get penalized by a lower single utility score *Z*_1_, which translates into a lower tolerance with respect to the targeted best performance. This means that the Pareto solutions in the vicinity of the anchor point, where the best solution of the individual function *f*_1,max_ lies, get a higher score and better ranking. The effect of α_*i*_ can be summarized in Figure 10.

The score is strongly related to the parameter α_*i*_ which represents the tolerance of the objective function at hand to deviation from the best individual optimum, as shown. For instance, when α_2_ = 0.1, the score associated with individual utility function *Z*_1_ decreases slowly at the start and then drops sharply when f1 approaches its worst value. This represents the case when a deviation from the best solution (minimum shutdown time of ∼18 min) is tolerated, and as a result, the score remains high for most of the objective function values until the worst solution is approached in which case the score drops sharply. Conversely, when the value of α is very large, only the solutions close to the single optimum get larger scores, and a deviation from this neighborhood results in a sharp drop of the individual score which asymptomatically approaches 0 when approaching the worst solution. This behavior is typically encountered in cases where solutions as close as possible to the individual optimum are prioritized, meaning the case of low or poor tolerance to deviation from the best individual solution.

The overall multiattribute utility function which allows the final ranking is obtained by combining the single utility functions in eq 29 which represent the individual scores.
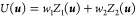
29where *w*_1_ and *w*_2_ represent the individual weights. And based on this method, the multi objectives are converted to one single score, which provides a list from the best to the worst operation alternatives based on the decision-making properties embedded into the MAUT.

**Figure 11:**
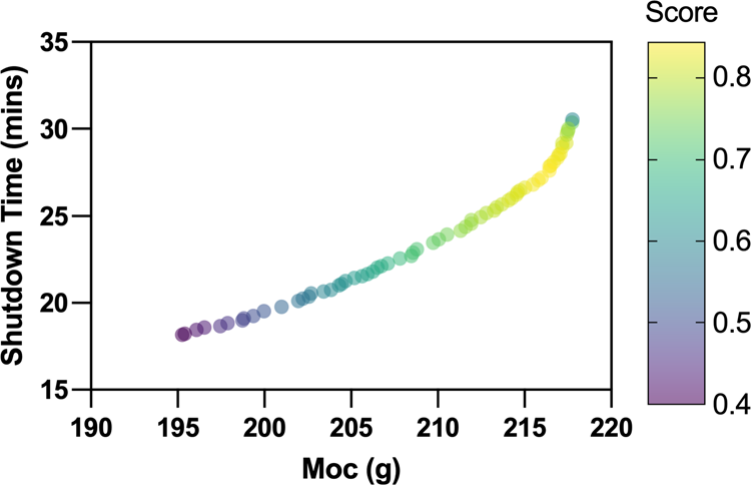
MAUT-based ranking of the Pareto solutions.

**Figure 12:**
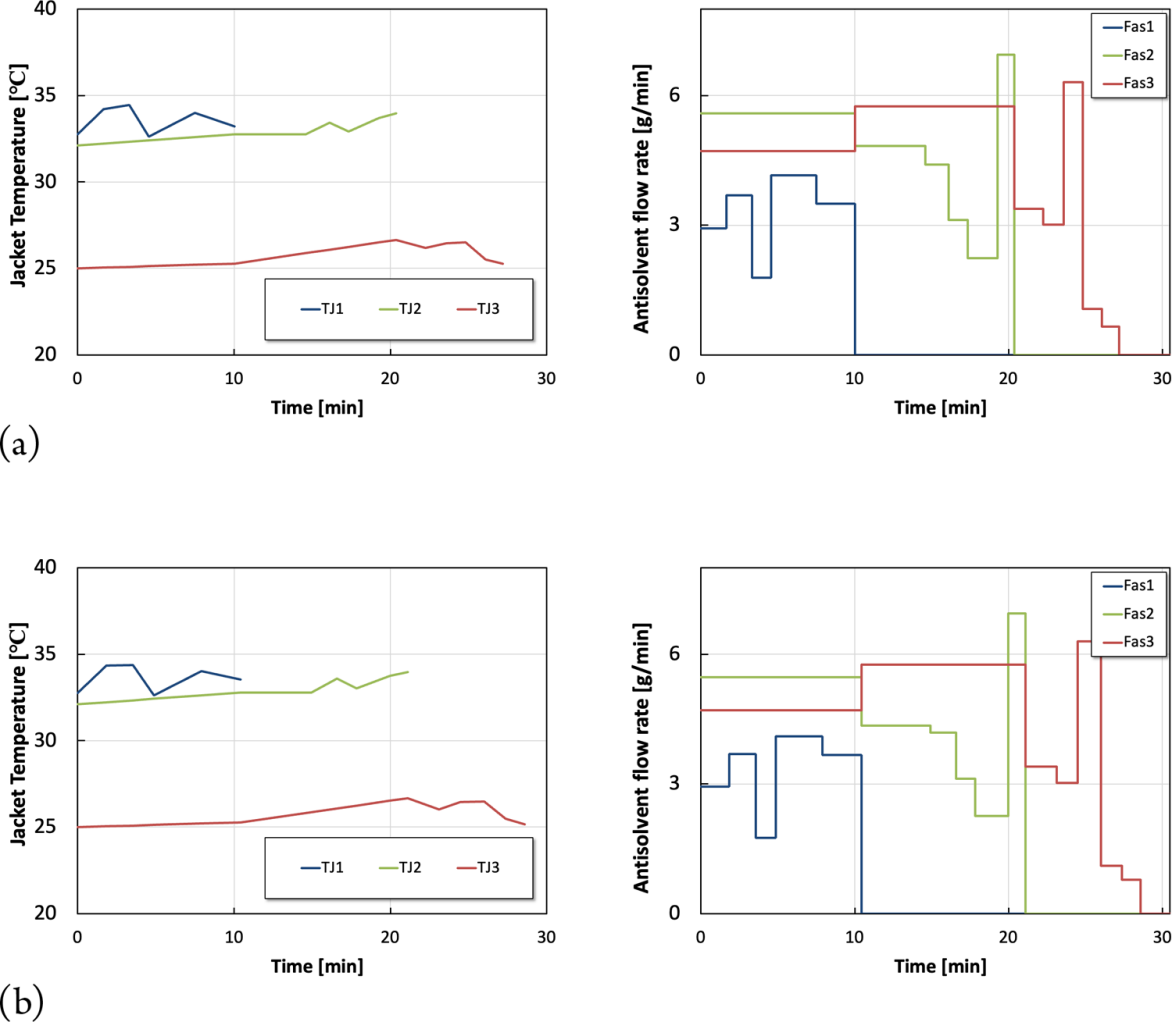
Optimal operating profiles of the 2 top ranking Pareto solutions. (a) Optimal jacket temperatures and antisolvent flow rates for the top-ranking solution. (b) Optimal jacket temperatures and antisolvent flow rates for the second top-ranking solution.

By using the weights and relative tolerance values outlined in Table 3, the Pareto solutions of the MOO problem were ranked, as shown in Figure 11. The total scores are captured by the different colors. Using MAUT, two top solutions, *S*1 and *S*2, can be clearly identified with scores exceeding 0.84. These two solutions correspond respectively to a maximum on-spec production of 215.97 and 217.05 g and a minimum shutdown time of 27.20 and 28.58 min. Compared to the remaining Pareto solutions, the top two solutions exhibit the best compromises between the shutdown time and on-spec production based on the selected multicriteria decision-aiding parameters. The optimal operating profiles of the two top-ranking Pareto solutions are depicted in Figure 12.

**Table 3:** Proposed Weights and Parameters of the Individual Utility Functions

objective function	weighting factor (*w*_*i*_)	α_*i*_
*Z*_1_ (shutdown time)	0.4	0.25
*Z*_2_ (mean crystal size)	0.6	1

The KPI scores associated with the two top-ranking Pareto solutions are summarized in Table 4. Overall, both solutions suggest similar operating strategies with minor differences. Similar KPI scores can be obtained with the top-ranking solutions, although *S*1 delivers shorter shutdown times, whereas *S*2 allows slightly more on-spec production, which highlights the competing nature of the Pareto solution. Most importantly, the top-ranking solutions (*S*1 and *S*2) obtained using a multiobjective optimization strategy clearly outperform scenario 4 discussed above, in the single objective optimization section, both in terms of shorter shutdown time and productivity.

**Table 4:** Key Performance Indicators Associated with the Top-Ranking Pareto Solutions

top raking solution	number of decision variables	shutdown time	total antisolvent added	residual material	E-factor	total on spec production	STSPR
*S*1	51	27.20	235.12	264.80	6.58	215.97	1.17
*S*2	51	28.58	235.71	264.31	6.55	217.05	1.12

## Conclusions

A series of new model-based optimal shutdown strategies were developed to convert residual material left at the end of the steady-state operation as waste into on-spec products. A three-stage cooling antisolvent continuous crystallization process was used to demonstrate the benefits of the proposed strategies. The idea is to identify optimal open-loop control profiles of the temperature and antisolvent addition at the different crystallization stages. First, five single objective optimization scenarios were considered to investigate the impact of the degrees of freedom (i.e., size of the decision vector), the discretization scheme, and computational cost as well as the impact of fixed and optimized shutdown time. The performance of the proposed scenarios was evaluated based on a set of environmental and economic KPIs. To allow a more effective evaluation of the shutdown performance against its steady state counterpart, a new KPI metric was introduced based on the shutdown to STSPR. It was shown that several optimization scenarios can deliver high productivity that matches or even exceeds the steady-state productivity. This result suggests that the throughput or productivity can be maintained effectively during the shutdown. More importantly, residual wastes and the E-factor can be significantly lowered while significant quantities of active pharmaceutical ingredients can be recycled back into the system. It was shown that increasing productivity may come at the expense of longer shutdown times, which dictated a new shutdown strategy based on multi-objective optimization. Consequently, a set of Pareto solutions that represent possible operating compromises was obtained. The top 2 ranking solutions identified using a multicriteria decision approach revealed enhanced productivity and shorter shutdown times compared to single objective optimization which paves the way for more effective shutdown operation based on both open-loop and closed-loop strategies.

## Supporting Information Available

All key inputs and parameters associated with the mathematical models of each of the crystallization stages; temperatures, antisolvent flow rates, mass, concentration, volumes, crystal size, and the moments associated with population balance models; and additional shutdown scenarios (PDF)

## Abbreviations

API: active pharmaceutical ingredient
ASA: aspirin, acetylsalicylic acid
CQA: critical quality attribute
CVP: control vector parametrization
DNC: direct nucleation control
KPI: key performance indicators
MSMPR: mixed suspension mixed product removal
PAT: process analytical technologies
SSC: supersaturation control
STSPR: steady state productivity ratio

## Notation

*B*_*i*_: nucleation rate at *i*th stage [cm^–3^/min]
*C**: solubility [g solute/g solvent mixture]
*C*: concentration [g solute/g solvent mixture]
*G*_*i*_: growth rate at *i*th stage [cm/min]
*F*_as,*i*,*j*_: antisolvent flow rate at the *i*th stage and *j*th time interval [g/min]
*F*_cr_: mass flow rate of on spec crystal [g/min]
*F*_feed_: feed flow rate [mL/min]
*k*_v_: volumetric shape factor
*L*: size of particles [cm]
*M*_ASA_: mass of ASA in *i*th stage [g]
*M*_AS_: mass of antisolvent in *i*th stage [g]
*M*_OS_: total mas of on spec product [g]
*M*_S_: mass of solvent in *i*th stage [g]
*n*_*i*_: number density at *i*th stage [cm^–3^]
*Q*_AS,*i*_: volumetric antisolvent flow rate at *i*th stage [cm^3^/min]
*Q*_E,*i*_: total net outlet flow rate of *i*th stage [cm^3^/min]
*Q*_in,*i*_: inlet flow rate of *i*th stage [cm^3^/min]
*Q*_out,*i*_: volumetric outlet flow rate at *i*th stage [cm^3^/min]
*S*: supersaturation at the *i*th stage [#]
*t*: time [min]
*t*_sd_: shutdown time [min]
*T*_J,*i*,*j*_: jacket temperature at the *i*th stage and *j*th time interval [°C]
*V*_*i*_: total volume at *i*th stage [mL]
*w*_*i*_: weight of *i*th objective
*Z*_*i*_: score of *i*th objective

## Greek Letter

μ_*k*,*i*_: *k*th moment at the *i*th stage
ρ_c_: density of the crystals [g cm^–3^]
ρ_*i*_: density of solution in *i*th stage [g cm^–3^]
ω_S_: solvent mass ratio [%]
ω_AS_: antisolvent mass ratio [%]
