# Supplementary material for: Single and Multiobjective Shutdown Optimization of a Multistage Continuous Crystallizer
Source: Ind Eng Chem Res. 2024 Apr 9;63(16):7300–14. doi: 10.1021/acs.iecr.3c03441 (PMC11046430; doi:10.1021/acs.iecr.3c03441)
Supplement: Supplementary file 1 — ie3c03441_si_001.pdf [file ie3c03441_si_001.pdf]

Supporting Information

Single and Multi-objective Shutdown Optimization of a Multistage Continuous Crystallizer

Jiaxu Liu[a], Brahim Benyahia[a*],

[a] *Chemical Engineering Department, Loughborough University, LE11 3TU Epinal Way, Loughborough, Leicestershire, UK*

[*]Email: b.benyahia@lboro.ac.uk

Table S1. Initial conditions obtained from steady state operation.

Variable	Stage 1	Stage 2	Stage 3
T(°C)	32.75	32.10	25
F_{as} (g/min)	9.94	5.88	2.68
Mass (solvent, antisolvent and API, g)	237.5	473.7	691.7
C (g/g solvent)	0.2475	0.1280	0.0640
V (ml)	203.5	414.4	609.9
d (μm)	225	353	417
μ_0(ml^{-1})	1370.16	1219.79	1190.91
μ_1(cm^{-2})	30.80	43.08	49.62
μ_2(cm^{-1})	1.38	2.37	2.99
μ_3	0.09	0.18	0.24

Table S1 summarizes all key inputs and parameters associated with the mathematical models of each of the crystallization stages. This includes temperature, antisolvent flowrate, mass, concentration, volume, crystal size, and the moments associated with population balance

models. These data capture the steady state conditions which essentially represent the initial conditions to the model-based optimal shutdown strategy.

Shutdown scenario 6

Scenario 6 was developed under the ideal assumption that all material present in each continuous crystallizer can be fully pumped out during the shutdown procedure. This suggests no loss of mixing efficiency while the vessel is being fully drained, and as result, no residue is left in the vessel at the end of the shutdown.

On-spec production was maximized by manipulating the jacket temperatures and antisolvent addition profiles based on a similar method as described in scenario 3. The corresponding mean crystal size trajectories are shown in figure S1.

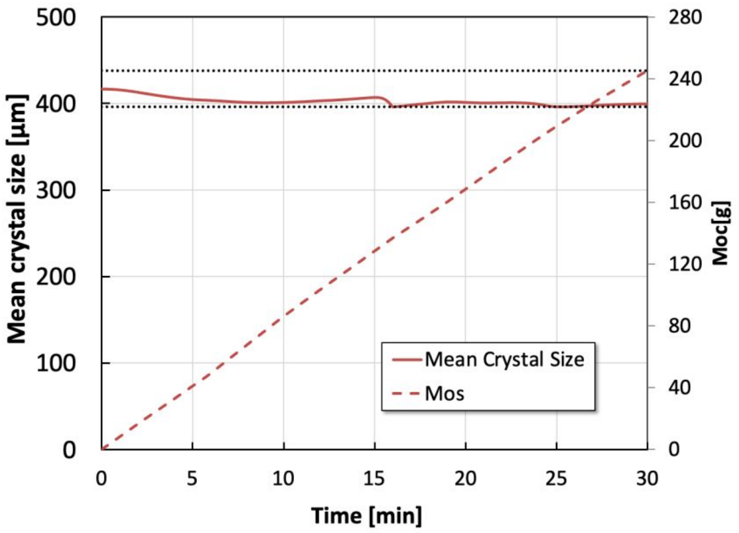

Figure S1. Optimal profiles of the mean crystal size and total on-spec crystals produced for the shutdown Scenario 6.

Shutdown scenario 7

In scenario 7, the three vessels were fully disconnected immediately at the beginning of the shutdown. The crystals present in third stage are on-spec and they are assumed to stay on spec while fed to downstream processing (e.g. wash-filtration). As such, the first and second stages were treated as semi-batch processes. By manipulating the antisolvent addition profiles and the jacket temperature profiles, the on-spec production can be maximized as summarized by the mathematical formulation in equation S1.

$$\underset{T_{J,i,j},F_{as,i,j}}{\boldsymbol{Max}} \; M_{os}(t_{sd})$$

s.t.

$$\dot{x} = f(x,y,u,p,t) \qquad x(t=0) = x_0$$

$$0 = g(x,y,u,p,t)$$

$$C1{:}0 \leq F_{as,i,j} \leq 20 \qquad \text{S1}$$

$$C2{:}25 \leq T_{J,i,j} \leq 40$$

$$C3{:}\omega_{as,i}(t) \leq 70\%$$

$$C4{:}S_i(t) \geq 1$$

$$i = 1,2$$

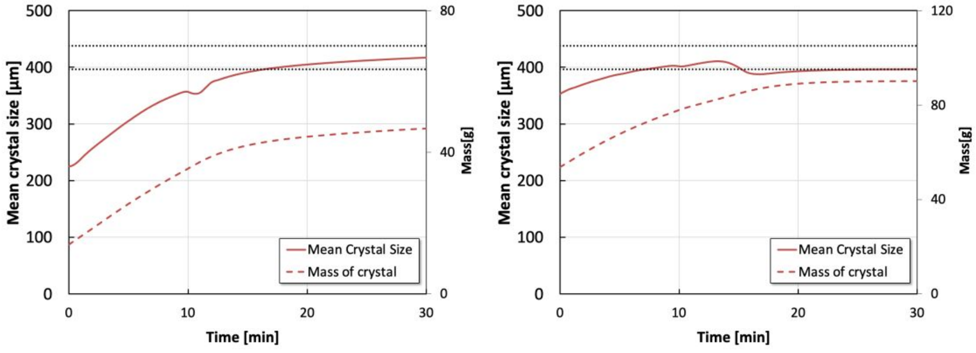

a b

Figure S2. Dynamic profiles of the mean crystal size total crystals produced for the shutdown Scenario 7, a) in the first crystallizer, b) in the second crystallizer.

Shutdown scenario 8

Scenario 7 was designed based on a reverse shutdown strategy string from third stage, second stage, then the first stage. The three vessels were treated as single stage continuous crystallizer until the shutdown procedure is complete. On-spec production was maximized by manipulating the jacket temperature profiles and antisolvent addition profiles. The optimal operation profile, the mean crystal size profile and total mass of on spec crystals produced are shown in figure S3.

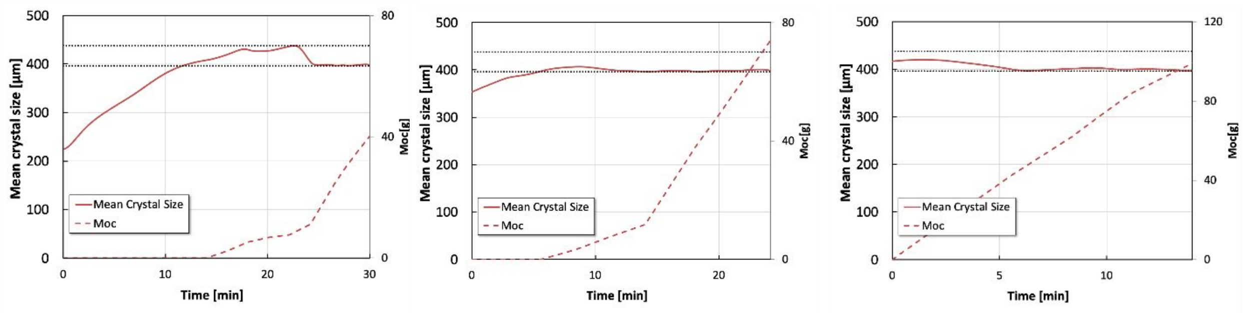

a b c

Figure S3. Dynamic profile of the mean crystal size and total mass of on spec crystals produced for scenario 7 in 1) a) stage 1, b) stage 2, and c) stage 3.

Table 2S. Summary of key performance indicators associated with the additional single objective shutdown optimization scenarios.

Scenarios	Number of decision variables	Shutdown time (min)	Total antisolvent added (g)	Residual material (g)	E-factor	Total on spec production (g)	STSPR
7	40	30	139.44	0	5.37	243.84	1.19
8	60	30	273.39	262.8	6.86	214.17	1.05
